# Explainable online health information truthfulness in Consumer Health Search

**DOI:** 10.3389/frai.2023.1184851

**Published:** 2023-06-21

**Authors:** Rishabh Upadhyay, Petr Knoth, Gabriella Pasi, Marco Viviani

**Affiliations:** ^1^Information and Knowledge Representation, Retrieval, and Reasoning (IKR3) Lab, Department of Informatics, Systems, and Communication, University of Milano-Bicocca, Milan, Italy; ^2^Big Scientific Data and Text Analytics Group, Knowledge Media Institute, The Open University, Milton Keynes, United Kingdom

**Keywords:** online health information, health misinformation, information truthfulness, information credibility, information retrieval, consumer health search, explainable artificial intelligence, explainable information retrieval

## Abstract

**Introduction:**

People are today increasingly relying on health information they find online to make decisions that may impact both their physical and mental wellbeing. Therefore, there is a growing need for systems that can assess the truthfulness of such health information. Most of the current literature solutions use machine learning or knowledge-based approaches treating the problem as a binary classification task, discriminating between correct information and misinformation. Such solutions present several problems with regard to user decision making, among which: (i) the binary classification task provides users with just two predetermined possibilities with respect to the truthfulness of the information, which users should take for granted; indeed, (ii) the processes by which the results were obtained are often opaque and the results themselves have little or no interpretation.

**Methods:**

To address these issues, we approach the problem as an *ad hoc* retrieval task rather than a classification task, with reference, in particular, to the Consumer Health Search task. To do this, a previously proposed Information Retrieval model, which considers information truthfulness as a dimension of relevance, is used to obtain a ranked list of both topically-relevant and truthful documents. The novelty of this work concerns the extension of such a model with a solution for the explainability of the results obtained, by relying on a knowledge base consisting of scientific evidence in the form of medical journal articles.

**Results and discussion:**

We evaluate the proposed solution both quantitatively, as a standard classification task, and qualitatively, through a user study to examine the “explained” ranked list of documents. The results obtained illustrate the solution's effectiveness and usefulness in making the retrieved results more interpretable by Consumer Health Searchers, both with respect to topical relevance and truthfulness.

## 1. Introduction

In recent years, several surveys have been conducted in different geographical areas worldwide that prove that people increasingly refer to information they find online to make decisions about their health (Akerkar et al., 2005; Fox and Duggan, 2013; Bahkali et al., 2016; Cao et al., 2016; Tran et al., 2021; Eurostat, 2022). The purposes for which people refer to such *Online Health Information* (OHI) (Thapa et al., 2021) are multifaceted and range from having support for decision making while still being guided by a medical expert (Powell et al., 2011), to making autonomous decisions based only on information retrieved firsthand (Tan and Goonawardene, 2017). It is clear that the latter behavior in particular can be very risky, especially when the information one is dealing with has not been generated or verified by physicians.

In the current context, in which anyone can generate and disseminate information online without employing official channels through a process known as *disintermediation* (Eysenbach, 2007), it is particularly necessary to have technological solutions available that can help people to juggle information of dubious truthfulness, particularly those persons with low *health literacy* (Graham and Brookey, 2008). According to Kindig et al. (2004), health literacy is “the degree to which individuals have the capacity to obtain, process, and understand basic health information and services needed to make appropriate health decisions”. In recent years, the problem of online *misinformation*, intended as incorrect or misleading information (Merriam-Webster, 2023b), has been addressed rather intensively, particularly with respect to *fake news* (Zhou and Zafarani, 2020) and *opinion spam detection* (Ferrara, 2019). More recent is the study of the problem with respect to the spread of so-called *health misinformation*, which we understand in this article as “a health-related claim of fact that is currently false due to a lack of scientific evidence” (Chou et al., 2018). Most of these literature solutions, regardless of the domain of reference, have attempted to limit the spread of misinformation through a binary classification task, that is, trying to identify truthful vs. deceptive information through the use of both supervised and/or unsupervised machine learning techniques or knowledge-based approaches.

While these types of solutions have proven to be effective with respect to the considered task and datasets (Viviani and Pasi, 2017; Zhou and Zafarani, 2020; Di Sotto and Viviani, 2022), it is not their purpose to consider critical issues from the perspective of the principles of so-called *Trustworthy Artificial Intelligence* (*Trustworthy* AI) (Chatila et al., 2021). Ethical issues that arise in this area concern the fact that the algorithms developed to identify misinformation should not impede *freedom of expression* and *autonomy* in decision-making. According to Brachman and Schmolze (1985), enabling AI systems to automatically control content would have a significant impact on the freedom of expression and information. This, in particular, because the frequency and conditions under which pre-screening or blocking occurs are uncertain (Marsden and Meyer, 2019), and because AI systems trained to detect misinformation could produce false positives and false negatives. Indeed, incorrect labeling of truthful content as misinformation by machines may result in an excess of censorship (Marsden and Meyer, 2019). Such problems, related to a non-transparent or incorrect identification or filtering of information judged as untruthful or deceptive, bring with it the problems related to awareness, and therefore autonomy, in decisions. One possible way to truly realize Trustworthy AI is to develop *Explainable* AI (XAI) solutions, especially in the area of health (Markus et al., 2021; Cabitza et al., 2022).

Hence, the purpose of this paper is precisely to address these issues in the context of ensuring user access to truthful information in the field of health. First of all, in order to avoid filtering information on the basis of its predicted truthfulness, the problem is studied in the context of the *Information Retrieval* (IR) task, and more specifically in that of *Consumer Health Search* (CHS), i.e., the search for health information by people without special medical expertise (Suominen et al., 2021). Additionally, the aim of this paper is to address the issue of *explainability of search results* in terms of information truthfulness. Explainability is crucial for users to understand why certain results are being presented to them, particularly in the case of health information where the consequences of acting on incorrect or misleading information can be severe (Harris, 2021).

The rest of the article is organized as follows: Section 2 summarizes basic concepts and key literature work with respect to issues tackled by this article; Section 3 illustrates the IR model used within CHS to obtain a ranked list of search results and describes the proposed solution to provide explainability to these results in terms of information truthfulness; Section 4 provides quantitative and qualitative assessments against the proposed solution and discusses them in detail; Finally, Section 5 concludes the article and defines some possible future research directions.

## 2. Background and related work

In this section, we introduce the concept of explainability in the context of Artificial Intelligence (Section 2.1) and we illustrate solutions tackling online (health) misinformation (Section 2.2). In particular, we focus on those solutions that are applicable to the delivery of explainable solutions for identifying truthful information in IR tasks, with special attention to the Consumer Health Search task. First, however, it is necessary to draw attention to the meaning of some concepts that we will use in the article. With regard to the concept of *truthfulness*, this term will serve to indicate correct information that tells the truth (Merriam-Webster, 2023c); for this purpose we understand it to be related to the concept of *factuality*, considering information that is supported by facts or evidence to be true (Merriam-Webster, 2023a). Accordingly, with regard to the concept of *health misinformation*, already introduced in Section 1, we again refer to information that is deceptive not being supported by scientific evidence (Chou et al., 2018).

### 2.1. Explainability in Artificial Intelligence

The concept of *eXplainable Artificial Intelligence* (XAI) refers to the ability of an AI system to provide clear and understandable explanations for its decision-making processes and outcomes (Adadi and Berrada, 2018; Guidotti et al., 2018). Similarly, according to Bansal et al. (2021), XAI addresses the challenge of understanding and interpreting the recommendations made by an AI model by generating explanations for its predictions. The *Defense Advanced Research Projects Agency* (DARPA) provides a wider definition of the purpose of XAI as to “produce more explainable models, while maintaining a high level of learning performance (prediction accuracy)”, and “enable human users to understand, appropriately trust, and effectively manage the emerging generation of artificially intelligent partners” (Gunning et al., 2019). From this latter definition, it emerges that explainability may increase *trust* in AI systems (Miller, 2019; Inam et al., 2021). In particular, according to Bjerring and Busch (2021), the ability to provide explanations for an AI's predictions increases the likelihood of people trusting the AI system and following its predictions.

One of the prevalent approaches to obtain XAI is to enhance system *transparency*. Transparency can be defined as the capability of a system to expose the reasoning processes behind its applications to the user (Gedikli et al., 2014). More specifically, transparency helps users understand systems' intentions, capabilities, and decision-making processes, which enhances the mutual understanding and awareness between users and the model (Bhaskara et al., 2020; Shin, 2021). For this reason, researchers and practitioners have been working to develop new techniques and methods to improve the transparency and, therefore, the explainability of AI systems. These efforts have included the development of *interpretable machine learning models*, as well as the use of *visualization tools* and other similar solutions, e.g., automatically generated *Natural Language Processing explanations*, to make the inner workings of AI systems more transparent (Bach et al., 2015).

### 2.2. Explainability in tackling online misinformation

A variety of approaches have been proposed in the last years to address the problem of the spread of misinformation online. Most formulate the problem as a *binary classification* task, distinguishing truthful information from misinformation, thereby possibly incurring the automatic information filtering problem discussed in the Introduction, and through techniques that often do not allow the user to fully understand how such a classification was generated (Viviani and Pasi, 2017; Islam et al., 2020; Zhou and Zafarani, 2020). Recently, some approaches have been developed to provide explanations for misinformation detection results. For example, in the fake news detection context, Shu et al. (2019) have developed an explainable fake news detection system that utilizes a co-attention mechanism in deep neural networks to capture explainable content in news articles and user comments. Lu and Li (2020) have proposed a graph-aware co-attention neural network scheme to generate explanations for fake news detection by analyzing user comments and retweet patterns on social media. Kou et al. (2020) have designed a graph neural network approach to detect and explain multi-modal fauxtography posts on social media. With regard to some approaches developed in the field of health, Ayoub et al. (2021) have proposed an explainable COVID-19 misinformation detection method that learns semantic representations of COVID-19 posts based on deep Natural Language Processing models, but only uses some words extracted from the posts for explanations. Kou et al. (2022) have designed a duo hierarchy attention-based approach, namely HC-COVID, that uses specific and generalized knowledge facts in a hierarchical crowd-source knowledge graph to explain COVID-19 misinformation effectively. However, these approaches to the explainability of results still apply to solutions that make a binary classification between information and misinformation.

For this reason, too, efforts are being made in recent years to address the problem of online misinformation by developing *Information Retrieval Systems* (IRS) that produce a ranked list of results that meet a user's information need while trying to uprank truthful results (Clarke et al., 2020; Pradeep et al., 2021; Suominen et al., 2021; Upadhyay et al., 2022). Such systems are relevant to our work as they do not produce a strict truthfulness judgment (used for binary classification), leaving the final decision to the user based on their investigation of the ranked list. Furthermore, this decision-making process can be complemented and supported by XAI solutions. Indeed, in the last bunch of years, there has been a growing interest in the field of *eXplainable Information Retrieval* (XIR), to improve the transparency of IR systems. While there are similarities between XIR and the broader field of XAI, there are also some notable differences due to the specific tasks, inputs, and output types involved in Information Retrieval, according to the classification provided by Anand et al. (2022). Three types of XIR solutions can be detailed: *post-hoc interpretability, interpretability by design*, and *grounding to IR principles*. *Post-hoc* interpretability involves providing explanations for decisions made by pre-trained machine learning models (Ribeiro et al., 2016; Lundberg and Lee, 2017). Several methods fall under this category, such as *feature attribution, free-text explanation*, and *adversarial example* methods. Feature attribution methods, also known as feature importance or saliency methods, generate explanations for an individual token by attributing the model output to input features (Qiao et al., 2019; Singh and Anand, 2019; Polley et al., 2021). On the other hand, free-text explanation methods provide explanations using natural language. Some methods are constituted by *point-wise explanations*, which use transformer-based models to generate free text explanations for individual query-document pairs (Rahimi et al., 2021); others are constituted by *list-wise explanations*, which use encoder-decoder transformers to generate text to explain all documents contained in a ranked result list for a given query (Yu et al., 2022). Lastly, adversarial example methods are commonly used to demonstrate the fragility or robustness of machine learning models and are typically used in classification tasks. However, in a retrieval task, the adversarial perturbation can be used to make a document rank higher or lower in the search results than it would. In the model proposed by Raval and Verma (2020), adversarial examples for black-box retrieval models are generated to lower the position of a top-ranked document using a stochastic evolutionary algorithm with a one-token-at-a-time replacement strategy. However, a challenge with *post hoc* interpretability methods is the difficulty in determining the extent to which the model behavior is understood. Rudin (2019) argued that interpretable-by-design models should be used as much as possible, especially for high-stakes decision-making situations. One way to increase the transparency of data-driven machine learning models is to determine if the trained models follow well-established IR principles. There are currently two research directions, (*i*) trying to align the predictions of ranking models with certain axioms; and (*ii*) examining the models to see if they incorporate known relevance factors such as matching, term proximity, and semantic similarity (Anand et al., 2022).

In light of these recent research directions, the solution proposed in this paper aims to provide explainable search results with respect to relevance factors (information truthfulness in particular) allowing users to gain a deeper understanding of the search process and the factors that influence the results (Yu et al., 2022), as illustrated in the next section.

## 3. Explaining information truthfulness in Consumer Health Search

This section is devoted to illustrating the solution proposed in this article to inject information truthfulness explainability into the search results obtained against the Consumer Health Search task. To do this, we refer to a previously proposed retrieval model that already takes into account information truthfulness (understood as illustrated at the beginning of Section 2) as a dimension of relevance in the ranking (described in Section 3.1) to which the explainability strategy proposed in this paper is applied (described in Section 3.2).

### 3.1. An IR model for truthful Consumer Health Search

In Upadhyay et al. (2022), we proposed a multidimensional retrieval model for CHS, i.e., a model to retrieve health-related documents by considering multiple relevance dimensions together. In this paper, we extend this model with an explainability component. This choice is motivated by several reasons, including: (*i*) the multidimensional nature of the IR model, so the resulting ranking may be difficult for users to understand; (*ii*) the fact that *information truthfulness* is the additional relevance dimension of documents beyond *topical relevance*, and it would be desirable to inform the user about the extent to which the provided documents are truthful; and (*iii*) the unsupervised nature of the model does not risk to introduce data bias in the generation of results, allowing us to overlook at least this problem.

In the Upadhyay et al. (2022) model, topical relevance is obtained by using the well-known and standard Okapi BM25 model (Brin and Page, 1998), which produces a *topicality score* denoted as BM25(*q, d*) for a query *q* and a document *d*. Formally:


(1) $$\begin{array}{l} \text{BM}25\left(q,d\right)=\underset{t\in q,d}{\sum }\log\frac{N-df\left(t\right)+0.5}{df\left(t\right)+0.5}\cdot \frac{tf\left(t,d\right)\cdot \left(k_{1}+1\right)}{tf\left(t,d\right)+k_{1}\cdot \left(1-b+b\frac{l_{d}}{L}\right)} \end{array}$$

The left side of the equation calculates the *Inverse Document Frequency* (IDF) of a term in relation to the entire document collection. Specifically, *N* represents the total number of documents in the collection, *df*(*t*) represents the document frequency for the term *t*, i.e., the number of documents containing *t*, *tf*(*t, d*) refers to the term frequency, i.e., the number of times *t* appears *d*. To account for differences in document length, length normalization is applied. *l*_*d*_ refers to the length *d*, *L* represents the average length of documents in the collection, and *k*_1_ and *b* are internal parameters used to adjust the scaling of term frequency and document length, respectively.

Information truthfulness is obtained by comparing the content of documents in the document collection and the content of scientific journal articles (considered reliable sources of evidence), both retrieved for the same query. The relevance of journal articles to queries is also calculated by means of the BM25 model. At this point, two BERT-based textual representation models are used to represent both documents and journal articles. One model is trained on MSMarco^1^, and the other on the BioBERT model, i.e., a pre-trained biomedical language representation model for biomedical text mining, trained on the *PubMed* and *PubMed Central* (PMC) datasets.^2^

Document and journal article textual representations are then compared by means of *cosine similarity*, to obtain an *information truthfulness score*. Formally:


(2) $$\begin{array}{l} its\left(q,d\right)=w_{1}\cdot \cos\left(d,j_{1}\right)+w_{2}\cdot \cos\left(d,j_{2}\right)+\ldots +w_{k}\cdot \cos\left(d,j_{n}\right) \end{array}$$

where *its*(*q, d*) refers to the information truthfulness score of the document *d* for a specific query *q*, *cos* is the *cosine similarity score*, and *w*_1_, *w*_2_, …, *w*_*k*_ are distinct weights assigned to each similarity score, such that ∑*w*_*i*_ = 1 and *w*_*i*_ ≥ *w*_*i*+1_ (1 ≤ *i* ≤ *k*−1). These weights allow assigning greater emphasis to the similarity scores according to the *rank* of the articles *j*_*j*_ retrieved with respect to *q*, as illustrated in detail in Upadhyay et al. (2022).

**Figure 1:**
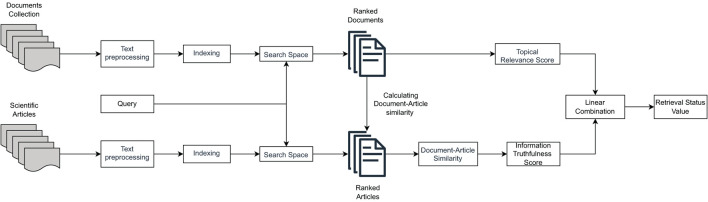
The retrieval model considering both topical relevance and information truthfulness (based on scientific evidence in the form of medical journal articles).

Finally, both topicality and information truthfulness scores are *linearly combined* to obtain the final *Retrieval Status Value* (RSV)—i.e., the global relevance score—for each document with respect to the considered query. Formally:


(3) $$\begin{array}{l} \text{RSV}\left(q,d\right)=w_{trs}\cdot \text{BM}25\left(q,d\right)+w_{its}\cdot its\left(q,d\right) \end{array}$$

where *w*_*trs*_ is an importance weight assigned to the topical relevance score, and *w*_*its*_ is an importance weight assigned to the information truthfulness score. They are used to decide whether to assign more importance in calculating the final value to topicality or truthfulness. Both weights assume values in the [0, 1] interval.^3^ Figure 1 illustrates the high-level operation of the multidimensional IR model under the CHS task proposed in Upadhyay et al. (2022).

While the above-mentioned retrieval model have proven to be effective compared to distinct monodimensional and multidimensional IR model baselines, it does not offer explanations to users with respect to how retrieved documents have been estimated to be truthful. However, we can add transparency to such a model by providing users with some information about the employed scientific evidence (i.e., factual information) supporting the claims in the retrieved documents in the search result page interface. This makes it possible to easily design a first proposal for the explainability of the truthfulness of search results on top of such a model.

### 3.2. Adding explainability for information truthfulness

**Figure 2:**
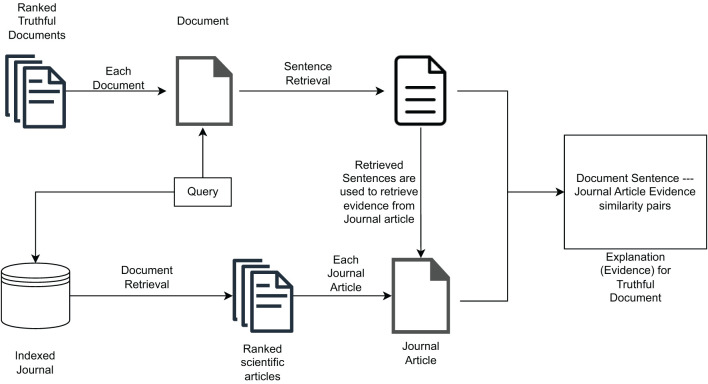
High-level outline of the scientific evidence extraction process to be provided to users.

The proposed solution aims at providing users with *scientific evidence* for truthfulness related to the ranking of documents produced by the model described in Section 3.1. To extract this evidence, as illustrated in Figure 2, we first retrieve *query-relevant passages* from retrieved documents, i.e., we identify portions of text in each document that are topically relevant with respect to the query. Then, we use these passages to extract *passage-based evidence* from journal articles that are topically relevant with respect to the query. Both query-relevant passages and passage-level evidence are then shown to users by means of a *Graphical User Interface* (GUI), which will be illustrated in detail in Section 4.3.1. This should help to increase the user's understanding of the obtained ranking and provide insight into the reasoning behind the truthfulness of each document in the ranked list.

#### 3.2.1. Extracting query-relevant passages from documents

**Figure 3:**
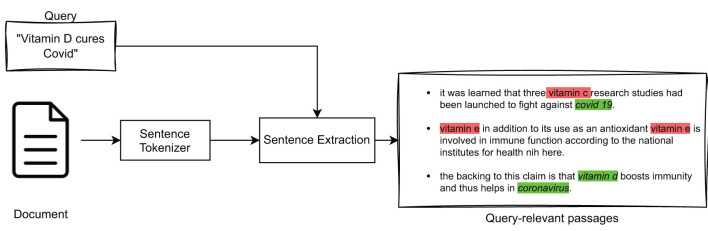
Query-relevant passage extraction.

This section details the process of extracting the most important passages from a document in relation to a given query. In fact, the IR model described in Section 3.1 returns a ranked list of documents, which were estimated to be “globally” relevant to a query. In our explainability model, we want to extract from such documents only those text passages that are topically relevant with respect to that query. For the purpose of this paper, one *sentence* was chosen as the size of a textual passage within a document. The high-level overview of this approach is illustrated in Figure 3.

Specifically, to extract *query-relevant passages* (sentences) we considered several strategies. The first strategy is based on representing queries and sentences as TF-IDF vectors, whose similarity is calculated by means of *cosine similarity*, according to which the sentences are ranked. The second strategy is based on using the BM25 model to obtain a ranked list of sentences relevant to the query. The last strategy involves the use of BioBERT to represent queries and sentences and again the use of cosine similarity to obtain a ranked list of sentences. In particular, BioBERT is a leading-edge language model in the biomedical field (Lee et al., 2020). It has proven to be particularly effective in various Natural Language Processing tasks related to medical texts, including *Question-Answering* (QA) (Poerner et al., 2020; Das and Nirmala, 2022) and *Named Entity Recognition* (NER) (Bhatia et al., 2019; Liu et al., 2021).

**Figure 4:**
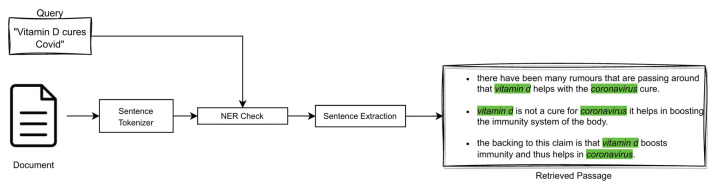
Query-relevant passage extraction with NER.

In particular, NER is a process of identifying *Named Entities*, i.e., real-world entities, such as people, organizations, places, dates, and more, in unstructured text. It can improve the sentence extraction process by providing context and additional information about such entities mentioned in medical sentences. Indeed, in medical texts, Named Entities play a crucial role in answering a query exactly; e.g., if the query is about “vitamin C” it would be incorrect to return a sentence that contained “vitamin D,” no matter how similar the two vector representations may be. For this reason, it was decided to incorporate NER in the three query-relevant passage extraction models considered (i.e., TF-IDF, BM25, and BioBERT), as high-level illustrated in Figure 4.

In particular, we compared the two Named Entities *medication*, denoted as μ, and *disease*, denoted as δ, present in both the query and the considered sentences. In this way, the similarity score between a query and a sentence (obtained either by means of cosine similarity or the BM25 similarity) has been modified so as to decrease it in the absence of correspondence between Named Entities. Formally:


(4) $$\sigma (q,s)=\{\begin{array}{ll} sim(q,s), & {\text{if NER}}_{q}(\mu ,\delta )={\text{NER}}_{s}(\mu ,\delta )\\ w_{d}\cdot sim(q,s), & \text{otherwise} \end{array}$$

where σ(*q, s*) indicates the similarity score between the query *q* and a sentence *s*, *sim*(*q, s*) indicates the similarity function employed to compute σ(*q, s*), which can be either cos(*q, s*) or BM25(*q, s*) depending on the employed model, NER_*x*_(μ, δ) indicates the Named Entities extracted from *x* (*x*∈{*q, s*}), and *w*_*d*_ (*w*_*d*_ ∈ [0, 1]) is a discount weight^4^, employed to decrease the value of σ(*q, s*) in the case of non-corresponding Named Entities in *q* and *s*.

#### 3.2.2. Extracting passage-based evidence from journal articles

After extracting the query-relevant passages from the documents, the step described in this section involves identifying, within scientific journal articles, pieces of *passage-based evidence* that support the query-relevant passages. This operation is performed on the scientific articles that had been identified as “globally” relevant to the query by the IR model shown in Section 3.1.

**Figure 5:**
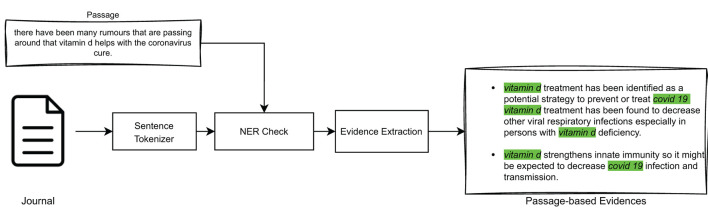
Evidence extraction using NER.

This can be achieved by using the same models illustrated in Section 3.2.1 for query-related passages extraction, i.e., models based on TF-IDF, BM25, or BioBERT, in association with Named Entity Recognition, and as high-level illustrated in Figure 5.

## 4. Experimental evaluations

Experimental evaluations were carried out with respect to two aspects. First, we evaluated from the *quantitative* point of view whether taking into account query-relevant passages and passage-based evidence actually allows for the identification of truthful documents. Second, we assessed from the *qualitative* point of view the effectiveness of our proposal with respect to the explainability of the results to users, by means of a user study involving human assessors.

### 4.1. The TREC “health misinformation track” dataset and technical details

The data used to implement and test the proposed solution are a subset of the TREC 2020 “Health Misinformation Track” dataset (Clarke et al., 2020). The track aims to encourage research on retrieval methods that “promote reliable and accurate information over misinformation in health-related decision-making tasks”. The original dataset is based on *CommonCrawl news*^5^, sampled from January 1st, 2020 to April 30th, 2020, and contains health-related news articles from around the world. Due to computational limitations, we randomly selected 219,245 English news articles related to COVID-19 from the original dataset, which is however unbalanced with a significantly higher number of negative samples compared to positive samples.

The dataset has a fixed structure, organized into *topics*, each of which includes a *title*, a *description* that reformulates the title as a question, a *yes/no answer* that is the actual answer to the description field based on the provided evidence, and a *narrative* that describes helpful and harmful documents in relation to the given topic. For example, the topic title: “ibuprofen COVID-19” has as description: “Can ibuprofen worsen COVID-19?”, as a yes/no answer: “no”, and as a narrative: “Ibuprofen is an anti-inflammatory drug used to reduce fever and treat pain or inflammation”.

The considered dataset also includes an evaluation set of 5,340 labeled data. The data are labeled with respect to *usefulness, answer*, and *credibility*. Usefulness corresponds to topical relevance; the answer indicates whether the document provides an answer to the query contained in the description field; credibility, in the context of this work, is employed as an approximation of a truthfulness label.^6^ In this paper, we considered only the usefulness and credibility labels, which are given on a binary scale, i.e., useful or not useful and credible or not credible.

To index documents and apply BM25-based retrieval models, we employed the BM25 implementation provided by *PyTerrier* (version 0.7.0)^7^ with default parameters. To retrieve documents, we used the description of the topic in the considered TREC 2020 dataset as the query. The same procedure was also adopted to find the journal articles related to the considered query, which were used to extract pieces of evidence (explanations). The adopted BioBERT model was dmis-lab/biobert-v1.1^8^, which employs sentence-transformers and is pre-trained on biomedical data (Lee et al., 2020). For tokenizing passages (i.e., sentences in our solution), we used the *Python* implementation of the sent_tokenise method provided by the *Natural Language Processing Toolkit* (NLTK version 3.8).^9^ For Implementation of Graphical User Interface, we used *anvil*^10^, as it is a web app building framework that is easy to use and deploy. For all of our experiments, we used *Python* (version 3.7).

### 4.2. Quantitative evaluation of effectiveness

The objective of quantitative model evaluation is to determine whether the similarity scores between query-relevant passages and passage-based evidence pieces are effective in identifying the truthfulness of retrieved documents with respect to a query. Indeed, the purpose of the article is to provide explanations to users based on such similarity, so they must also prove effective with respect to the task of identifying truthful information as a whole.

Hence, the similarity scores between a query-relevant passage and pieces of passage-based evidence were computed, and this score was employed to classify documents as truthful or non-truthful. In particular, we considered two solutions to calculate the final similarity score between the query-relevant passage and pieces of passage-based evidence. The first solution considers the similarity scores between the query-relevant passage and different pieces of passage-based evidence and calculates their *mean*. The second solution considers the *maximum* similarity score between the query-relevant passage and the different pieces of passage-based evidence as the similarity score.

With respect to both models, we tested query-relevant passage and passage-based evidence extraction models based on TF-IDF, BM25, and BioBERT, with and without the application of NER. We performed the experiments considering a variable number of retrieved documents (i.e., 10, 20, 50, and 100), a variable number of retrieved scientific journal articles (i.e., 1, 5, and 10), a variable number of query-relevant passages extracted from the documents (i.e., 5 and 10). In all cases, the number of pieces of passage-based evidence taken into account was equal to 5.

In performing experiments with respect to each of these configurations, we applied *five-fold cross-validation* in the following way. Query and document pairs were randomly and independently divided into five folds, with each fold containing a subset of the total data. In each iteration of the cross-validation process, one fold was used as the test set, while the other four folds were used as the training set to train the model. The model was then used to calculate the similarity score between the query-relevant passages extracted from the documents and the pieces of passage-based evidence from the journals, based on the considered queries. This approach allowed us to evaluate model performance using all available data, while ensuring that the evaluation was not biased by using different subsets of data for training and testing in each iteration of the cross-validation process. Performance was evaluated in terms of F1 score (F1), *Geometric Mean* score (GM), commonly used for imbalanced datasets (Davagdorj et al., 2020), and *Area Under the* ROC *Curve* (AUC).

Tables 1, 2 show the model performance using the BioBERT model for both query-relevant passage and passage-based evidence extraction, without and with the application of NER. Standard deviation values for all the results presented in the table are between ±0.01 and ±0.03. The presented results are averaged results for each fold under each parameter configuration. In the tables, the column “#docs” indicates the number of considered retrieved documents, “#journals” the number of considered journal articles, and “#doc-passages” the number of retrieved passages per document. The section indicated by “mean-similarity” shows the results obtained by computing the mean similarity among the retrieved query-relevant passages and pieces of passage-based evidence, while the “max-similarity” section presents the result obtained by considering the highest similarity score among them.

**Table 1:** Quantitative evaluations of the BioBERT model without NER.

**Description**	**#docs**	**#journals**	**F1**	**GM**	**AUC**	**F1**	**GM**	**AUC**
			**#doc-passages = 10**			**#doc-passages = 5**		
	**Mean-similarity**							
BioBERT w/o NER	100	10	0.61	0.55	0.52	0.6054	0.534	0.507
		50	10	0.613	0.54	0.53	0.613	0.543	0.5871
		20	10	0.601	0.53	0.587	0.6401	0.532	0.581
		10	10	0.64	0.54	0.579	0.6398	0.534	0.596
		100	5	0.604	0.5503	0.521	0.631	0.557	0.512
		50	5	0.601	0.549	0.567	0.634	0.534	0.533
		20	5	0.619	0.543	0.594	0.667	0.545	0.601
		10	5	0.625	0.521	0.59	0.6465	0.556	0.578
		100	1	0.634	0.567	0.556	0.645	0.567	0.534
		50	1	0.654	0.538	0.567	0.634	0.534	0.564
		20	1	0.698	0.534	0.6013	0.688	**0.584**	0.607
		10	1	0.7	0.546	0.581	0.6742	0.534	0.587
		**Max-similarity**							
		100	10	0.623	0.54	0.54	0.612	0.56	0.53
		50	10	0.63	0.546	0.544	0.632	0.546	0.546
		20	10	0.678	0.571	**0.613**	0.687	0.546	**0.624**
		10	10	0.672	0.578	0.608	0.647	0.567	0.617
		100	5	0.634	0.5467	0.534	0.612	0.545	0.53
		50	5	0.64	0.567	0.545	0.638	0.565	0.546
		20	5	0.678	0.557	0.624	0.645	0.53	0.617
		10	5	0.657	0.566	0.614	0.641	0.534	0.601
		100	1	0.6533	0.566	0.546	0.564	0.567	0.536
		50	1	0.655	0.546	0.534	0.645	0.567	0.567
		20	1	**0.703**	**0.589**	0.607	**0.698**	0.557	0.614
		10	1	0.695	0.59	0.6	0.687	0.587	0.598

Bold values represent best results.

**Table 2:** Quantitative evaluations of the BioBERT model with NER.

**Description**	**#docs**	**#journals**	**F1**	**GM**	**AUC**	**F1**	**GM**	**AUC**
			**#doc-passages = 10**			**#doc-passages = 5**		
	**Mean-similarity**							
BioBERT w NER	100	10	0.66	0.581	0.56	0.6565	0.5801	0.566
		50	10	0.66	0.574	0.58	0.655	0.573	0.5871
		20	10	0.66	0.57	0.63	0.6901	0.583	0.633
		10	10	0.67	0.58	0.62	0.6701	0.581	0.623
		100	5	0.66	0.5801	0.564	0.664	0.582	0.559
		50	5	0.665	0.574	0.581	0.6547	0.573	0.581
		20	5	0.6852	0.585	0.624	0.694	0.585	0.631
		10	5	0.6816	0.585	0.616	0.6858	0.586	0.614
		100	1	0.684	0.588	0.576	0.6733	0.584	0.573
		50	1	0.684	0.582	0.597	0.667	0.576	0.588
		20	1	0.7107	0.593	0.6313	0.728	**0.603**	0.627
		10	1	0.7129	0.597	0.607	0.7042	0.594	0.602
		**Max-similarity**							
		100	10	0.6568	0.58	0.56	0.655	0.58	0.558
		50	10	0.66	0.575	0.584	0.661	0.574	0.584
		20	10	0.707	0.591	**0.643**	0.696	0.586	**0.644**
		10	10	0.702	0.592	0.639	0.673	0.582	0.638
		100	5	0.6524	0.5798	0.552	0.652	0.579	0.55
		50	5	0.67	0.577	0.579	0.659	0.574	0.579
		20	5	0.698	0.587	0.639	0.679	0.58	0.639
		10	5	0.677	0.583	0.633	0.695	0.588	0.628
		100	1	0.6733	0.584	0.569	0.674	0.584	0.566
		50	1	0.679	0.579	0.593	0.675	0.578	0.591
		20	1	**0.7334**	**0.606**	0.637	**0.722**	0.599	0.636
		10	1	0.74	0.619	0.622	0.711	0.597	0.619

Bold values represent best results.

As mentioned earlier, these results are higher than those using the TF-IDF and BM25 models in extracting query-relevant passages and passage-based evidence. For the sake of conciseness, in Tables 3, 4, we illustrate the results for these other two models compared to BioBERT only with respect to the best parameter configuration.

**Table 3:** Quantitative evaluations of the TF_IDF, BM25, and BioBERT models without NER.

**Description**	**#docs**	**#journals**	**F1**	**GM**	**AUC**	**F1**	**GM**	**AUC**
			**#doc-passages = 10**			**#doc-passages = 5**		
TF_IDF	20	1	0.6335	0.4823	0.4684	0.6337	0.4697	0.4433
BM25	20	1	0.6745	0.5131	0.5337	0.6701	0.5019	0.5195
BioBERT w/o NER	20	1	**0.703**	**0.589**	**0.607**	**0.698**	**0.557**	**0.614**

Bold values represent best results.

**Table 4:** Quantitative evaluations of the TF_IDF, BM25, and BioBERT models with NER.

**Description**	**#docs**	**#journals**	**F1**	**GM**	**AUC**	**F1**	**GM**	**AUC**
			**#doc-passages = 10**			**#doc-passages = 5**		
TF_IDF	20	1	0.6545	0.4998	0.4777	0.6443	0.4923	0.4663
BM25	20	1	0.6985	0.5432	0.5542	0.6881	0.5213	0.5305
BioBERT w NER	20	1	**0.7334**	**0.606**	**0.637**	**0.722**	**0.599**	**0.636**

Bold values represent best results.

Overall, we can observe that the BioBERT model, both with and without the application of NER, outperforms all other models in terms of F1 score, GM, and AUC. Furthermore, incorporating NER generally improves the performance of the models across the board. In addition, the BioBERT model with NER achieves the highest F1 score, GM, and AUC, indicating better performance in misinformation detection with respect to other baselines.^11^ We also note that the “max-similarity” model performs better than the “mean-similarity” model. It is also clear from the tables that the application of NER leads to a significant increase in performance, enabling more accurate identification and retrieval of topically relevant sentences that contain important entities or concepts related to the query and evidence from the journal articles. In general, with respect to effectiveness in classifying health misinformation, using only some of the passages and not the whole document, we can see that classification performance, especially in terms of F1 score, can be considered quite satisfactory as a preliminary result although not exceptional, considering that the classification of misinformation is not the purpose of the article. We must also remember that there may be a potential decoupling between the concept of truthfulness used in this article and the concept of credibility that was used as a classification label in the dataset under consideration, in the absence of other datasets useful for the purpose in the health domain.

### 4.3. Qualitative evaluation of effectiveness

The objective of the qualitative model evaluation is to understand the effectiveness of the proposed explainability strategy by assessing the usefulness of the information and scientific evidence provided to users by means of a *user study*. This can help improve the proposed model and guide the development of additional tools or techniques to improve the explainability of the results obtained by means of the model.

The user study was conducted with 18 human assessors, all doctoral and master's students experienced in NLP and IR, respecting the age and gender balance criteria. The study was performed by means of a specifically-designed *Graphical User Interface* (GUI). Assessors were given clear guidance on the domain under consideration, how to use the GUI, and what aspects to evaluate.

In the following, the GUI is detailed in Section 4.3.1. By means of this GUI, the users were required to perform some *tasks*, illustrated in Section 4.3.2. Later, users were required to answer a *questionnaire*, detailed in Section 4.3.3. Based on this questionnaire, it was possible to assess the *outcome* of user satisfaction with respect to the explainability of the results obtained, as discussed in Section 4.3.4.

#### 4.3.1. The Graphical User Interface

**Figure 6:**
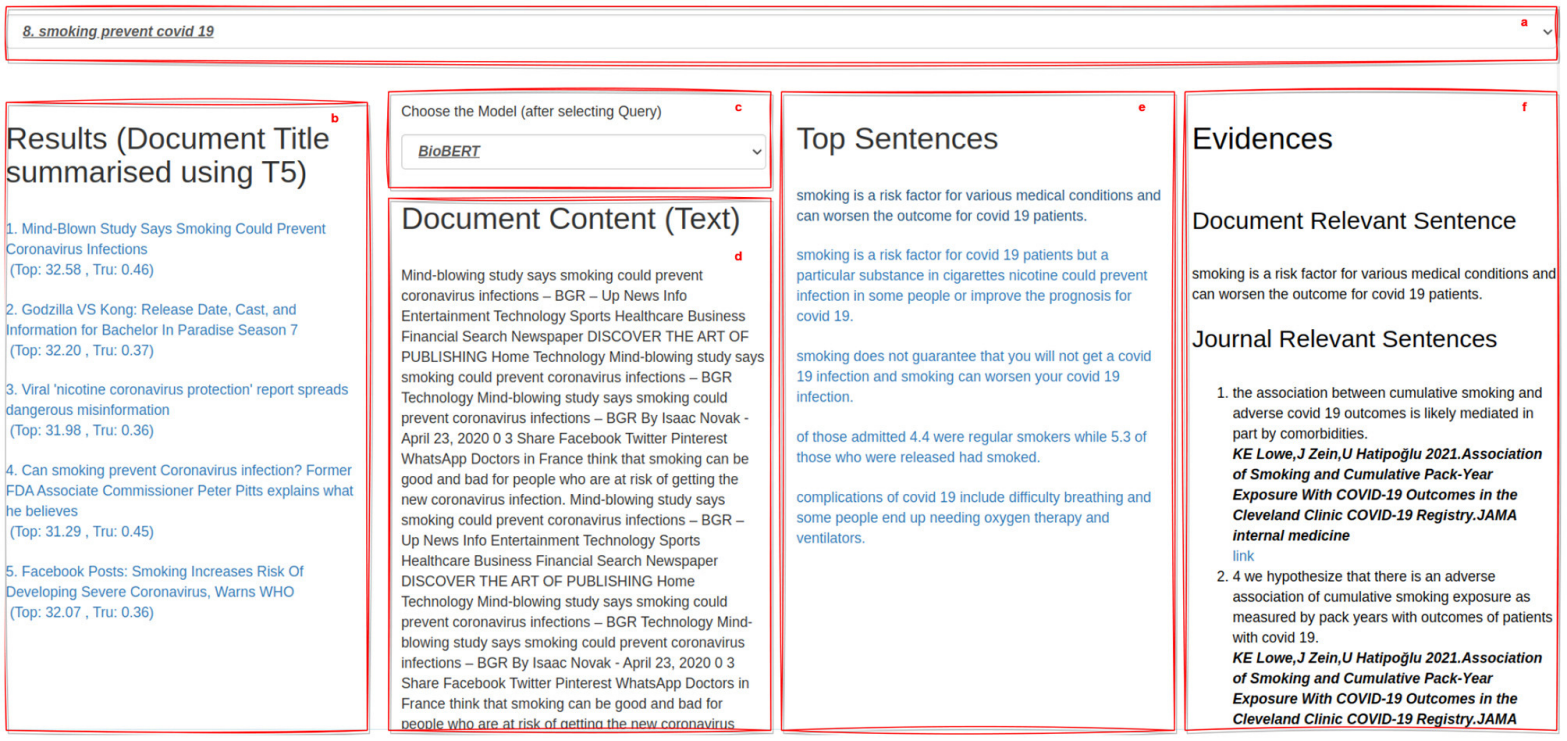
The Graphical User Interface.

The appearance of the developed GUI is illustrated in Figure 6.

Here are visible some key components of the interface, which can be summarized into five main panels, as follows.

(*a*) *The Query Panel*: it presents the set of 12 randomly-chosen queries from those available in the dataset (i.e., 48) from which human assessors can choose;

(*b*) *The Ranking Panel*: it presents the ranked list of the top-5 documents retrieved w.r.t. a query, by using the IR model detailed in Section 3.1. In particular, in this panel, the title associated with these documents are presented. Since in the original dataset, no titles described the content of documents, we employed the T5 model over documents to produce significant titles.^12^ This ranked list further associates, to each document title, the document's topicality score (*Top*) and truthfulness score (*Tru*);

(*c*) *The Sentence Extraction Model Panel*: it allows human assessors to choose among the list of the three distinct models for extracting query-relevant passages and pieces of passage-based evidence in the form of sentences. As illustrated in Section 3.2.1, the three models are based on TF-IDF, BM25, and BioBERT;

(*d*) *The Document Content Panel*: by selecting a document from panel (*b*), this panel shows its content and highlights the query-relevant passages identified by using the sentence extraction model selected in the panel (*c*);

(*e*) *The Top Sentences Panel*: this panel illustrates the list of the query-relevant passages extracted for the query selected in panel (*a*), in the document selected in panel (*b*), for the extraction model selected in panel (*c*);

(*f*) *The Evidence Panel*: it shows human assessors pieces of passage-based evidence from journal articles for the selected sentence in panel (*e*).

#### 4.3.2. The tasks

The tasks were designed to have human assessors test different query-relevant passage and passage-based evidence extraction models to determine the best way to explain the truthfulness of a document. In particular, the human assessors were required to perform the following tasks.

(*i*) *Evaluate the Ranking*: in this task, assessors were asked to select a query from panel (*a*), analyze the documents in the obtained ranking against that query and the associated topicality and truthfulness scores in panel (*b*), and evaluate, based on these and the content of the retrieved documents, which dimension of relevance they believed had the greatest impact on the final ranking;

(*ii*) *Evaluate the Query-relevant Passages*: in this task, assessors were asked to evaluate, for each document returned in the ranking based on the query chosen in panel (*a*), what was the best query-relevant passages extraction model. To do this, each assessor had to first choose a document from the ranking in panel (*b*), choose a model from panel (*c*), and analyze the highlighted sentences in panels (*d*) and (*e*);

(*iii*) *Evaluate the Passage-based Evidence*: in this task, assessors were asked to evaluate, for each query-relevant passage in panel (*e*) extracted from each document found in panel (*b*) against the query in panel (*a*) and the model chosen in panel (*c*), the usefulness and reliability of the scientific evidence associated with each step and illustrated in panel (*f*), to determine whether the supporting scientific evidence was sufficient and clear to determine the truthfulness of the document.

#### 4.3.3. The questionnaire

The questionnaire was used to collect information on the perceived quality of both query-relevant passage and passage-based evidence extraction models and to understand the users' level of satisfaction with the explainability of the truthfulness of the retrieved documents. In particular, the questionnaire contains a set of questions related to panel (*b*): for assessing the clarity and influence of topicality and truthfulness in the ranking of the document; to panels (*c*), (*d*), and (*e*): for finding the best method to retrieve sentences and to understand the effectiveness of this choice; and to panels (*c*), (*e*) and (*f*): for assessing the usefulness and quality of evidence provided.

Questions related to ranking—panel (*b*)—are as follows:

− *Are topicality and truthfulness scores useful to understand the ranking?*

− *Do you think this ranking is more influenced by topicality or truthfulness?*

Questions related to sentence extraction (query-relevant passages)—panels (*c*), (*d*), and (*e*)—are as follows:

− *Are the highlighted sentences topically related to the query by using either TF-IDF, BM25 or BioBERT?*

− *Which of the three models best captures the previous aspect?*

− *Do the highlighted sentences (with the best model between TF-IDF, BM25, or BioBERT) provide sufficient information to determine the truthfulness of the document?*

− *Do you think highlighting a single sentence is enough to capture both the topicality and truthfulness of the document?*

Questions related to sentence extraction (passage-based evidence)—panels (*c*), (*e*), and (*f*) are as follows:

− *Are the top sentences (with the best model between TF-IDF, BM25, or BioBERT) correctly supported by scientific evidence (scientific journal articles)?*

− *Does the scientific evidence provide sufficient information to assess the truthfulness of the document?*

− *Do you think the information sources (the scientific journal articles) associated with each highlighted sentence are trustworthy?*

Some questions in the questionnaire were designed in a way that allows participants to answer *yes, no, don't know*, or *other*. Some questions in the questionnaire include multiple-choice questions that allow participants to choose specific methods or ways for document ranks, extracted sentences, and evidence.^13^

#### 4.3.4. Outcome of the questionnaire

The responses to the questionnaire were collected and analyzed to gain insights into the users' perspectives on the proposed model. Given our 18 human assessors, a total of 36 responses were gathered, with three responses per question. In particular, to evaluate the *inter-rater reliability* of the study, we computed *Fleiss' kappa measure* (Fleiss, 1971) for each question as rated by three raters. Fleiss' kappa quantifies the level of agreement among multiple raters, with values closer to 1 indicating stronger agreement. Table 5 displays the mean Fleiss' kappa values for each question across all questions.

**Table 5:** Mean Fleiss' kappa score for each question for 3 raters.

**ID & Category**	**Question**	**Fleiss' kappa**
Q1. *Ranking*	*Are topicality and truthfulness scores useful to understand the ranking?*	0.85
Q2. *Ranking*	*Do you think this ranking is more influenced by topicality or truthfulness?*	0.65
Q3. *Passage extraction*	*Are the highlighted sentences topically related to the query by using either TF-IDF, BM25 or BioBERT?*	0.88
Q4. *Passage extraction*	*Which of the three models best captures the previous aspect?*	0.89
Q5. *Passage extraction*	*Do the highlighted sentences (with the best model between TF-IDF, BM25, or BioBERT) provide sufficient information to determine the truthfulness of the document?*	0.70
Q6. *Passage extraction*	*Do you think highlighting a single sentence is enough to capture both the topicality and truthfulness of the document?*	0.64
Q7. *Evidence extraction*	*Are the top sentences (with the best model between TF-IDF, BM25, or BioBERT) correctly supported by scientific evidence (scientific journal articles)?*	0.85
Q8. *Evidence extraction*	*Does the scientific evidence provide sufficient information to assess the truthfulness of the document?*	0.78
Q9. *Evidence extraction*	*Do you think the information sources (the scientific journal articles) associated with each highlighted sentence are trustworthy?*	0.91

Overall, the table shows fairly high Fleiss' kappa scores, ranging from a low of 0.64 to a high of 0.91, indicating a satisfactory to high level of agreement among assessors for each question.

##### Q1–Q2. Ranking

**Figure 7:**
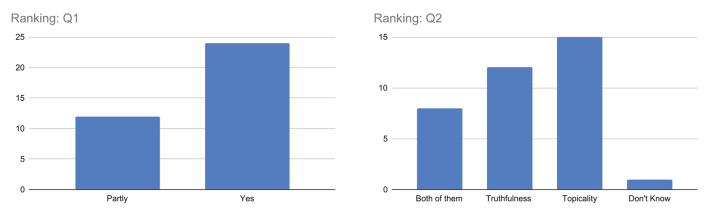
Outcome of the questions related to ranking.

From Figure 7, when considering question Q1, it is interesting to note that the majority of the respondents answered *yes*, indicating that they consider the visualization of both topicality and truthfulness scores to be useful in understanding the obtained ranking. However, there is also a non-negligible number of respondents who answered *partly*, suggesting that some respondents may not fully understand the concepts or how they are related to the ranking.

Regarding question Q2, there is more variability in the responses, with approximately equal numbers of respondents choosing *topicality* and *truthfulness* as the factors having the greatest impact on ranking. This suggests a different perception of the respondents with respect to the importance of these factors in the process of ranking the results, which needs to be investigated more in the future also considering the psychological aspects of assessors. However, the limited number of responses *don't know* indicates that only a few respondents fail to get an idea of which dimension of relevance is actually most important with respect to the results obtained.

##### Q3–Q6. Passage extraction

**Figure 8:**
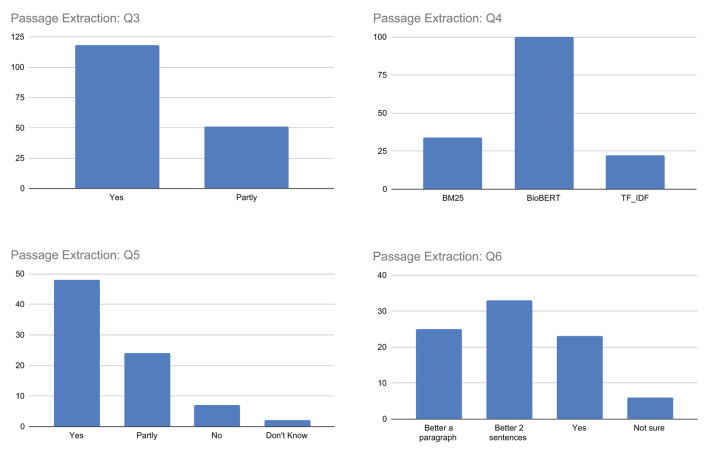
Outcome of the questions related to query-relevant passage extraction.

The results for question Q3, as illustrated in Figure 8, show that most respondents answered with *yes*, indicating that the highlighted sentences were mostly considered topically related to the query using the TF-IDF, BM25, or BioBERT models. However, it is worth noting that the responses were not entirely unanimous, with some users responding with *partly*, suggesting that there may be room for improvement in accurately identifying and extracting the most relevant passages. Ultimately, user responses offer valuable insights that can guide future improvements to the proposed model, in particular when analyzing the replies to the next questions.

In response to question Q4, which asks users to identify the best algorithm for topicality-based passage extraction between TF-IDF, BM25, and BioBERT, the majority of users choose BioBERT. This is illustrated in Figure 8. However, there is a non-negligible number of users who chose the other algorithms, suggesting also in this case some perception differences among assessors, maybe due to specific queries and/or documents.

Regarding question Q5, responses were mixed, with the maximum number of respondents answering affirmatively (*yes* or *partly*) and some answering negatively (*no* or *don't know*). This suggests that while the majority of users found the highlighted passages informative, others still did not consider them sufficient as meaningful sentences from which to determine the document's truthfulness. It is important to note that this question is complex, as it involves not only the (topical) relevance of the highlighted passages to the query, but also their ability to provide starting points for identifying evidence for or against the document's truthfulness.

Finally, when considering question Q6, which asks whether singling out single sentences as passages are sufficient to capture both topicality and truthfulness aspects of the document, the answers are quite varied. Users who believe that a single sentence is sufficient to capture both aspects are a minority. In general, most believe that a better approach would be to consider a passage consisting of more text, such as *two sentences* or *a paragraph*. This highlights the importance of considering such feedback in order to take into account a different granularity of text passages presented to users in the future.

##### Q7–Q9. Evidence extraction

**Figure 9:**
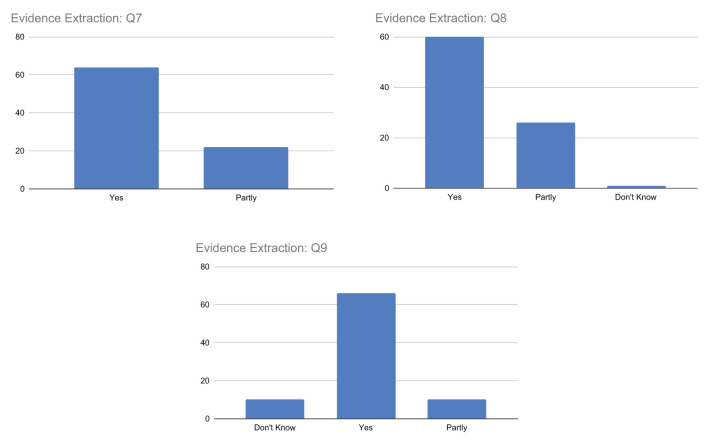
Outcome of the questions related to passage-based evidence extraction.

The replies associated with question Q7, summarized in Figure 9, generally indicate agreement among participants. However, while most respondents answered *yes*, indicating that the highlighted passages were supported by scientific evidence, a still significant number of respondents answered *partly*. This may suggest that not all highlighted passages are indeed fully supported by scientific evidence and/or that there may be a mix of both fully and partly supported passages.

Similarly, for question Q8, the majority of respondents answered *yes* indicating that the scientific evidence provided sufficient information to understand the document's truthfulness, while a significant number of respondents answered *partly*, suggesting that not all of the extracted evidence was fully helpful in determining the document's truthfulness. Some respondents also answered *don't know*. The responses suggest that scientific evidence can globally play a crucial role in supporting the explainability of the truthfulness of a document, even if for a small number of respondents this is not fully sufficient.

Finally, for question Q9, respondents expressed different opinions and uncertainties about the reliability of the sources associated with each piece of evidence. While a large percentage of respondents answered *yes*, indicating that they thought the sources were reliable, there were also some *partly* or *don't know* responses, suggesting uncertainty or lack of information on the part of the respondent. This may be due to the respondents' lack of health literacy to confidently assess the reliability of sources or the complexity or ambiguity of the question.

## 5. Conclusions and further research

In this article, we have presented a new approach to the explainability of search results in the context of Consumer Health Search, particularly regarding the truthfulness of the information. In particular, to provide interpretable results to users from this point of view, scientific evidence has been extracted from articles in medical journals that have been compared with relevant textual passages extracted from the documents retrieved with respect to the queries under consideration.

To carry out the extraction of (topically) relevant passages from documents and corresponding scientific evidence from scientific articles, we used various textual retrieval and representation techniques, with and without the aid of Named Entity Recognition techniques to consider the specificity of certain entities in the health domain. The proposed solution was evaluated both from a quantitative and qualitative point of view. The latter evaluation took place, in particular, by means of a user study, in which users were asked to perform tasks and answer a questionnaire.

Through this questionnaire, we were able to obtain valuable information from the assessors regarding their perception of the explainability of the results obtained. In particular, with respect to the ranking obtained and the effectiveness of the relevance dimensions, the extraction of textual passages from documents and scientific evidence from scientific articles and their usefulness in explaining why a document found was actually judged as satisfactory by the majority of respondents. We analyzed responses using the Fleiss' kappa score to assess the inter-rater reliability of the questionnaire and found that the level of agreement among raters was generally high.

However, the results of our user survey also revealed some limitations and room for improvement with respect to the proposed solution. For example, it appears that identifying textual passages in the form of single sentences within documents may not always be sufficient to provide a good starting point for assessing both the topical relevance and truthfulness of a document; some psychological factors of the users or other factors related to the dataset should be further investigated for a better understanding of the actual impact of the different dimensions of relevance; however, some assessors found it difficult to estimate the reliability of the information sources (and this is a problem closely related to health literacy); moreover, the quantitative evaluation has given encouraging albeit not excellent results. We, therefore, plan to address these limitations and further improve our approach in future work.

## Data availability statement

Code and documentation related to this original work can be found at the following link: https://github.com/ikr3-lab/explainableCHS-frontiers/. Further inquiries with respect to the data used and their availability can be directed to the corresponding author.

## Author contributions

RU, PK, GP, and MV contributed to the conception and design of the study. RU developed the software for model implementation and the Graphical User Interface and performed statistical analysis on quantitative results. RU and MV designed and followed the human assessor evaluation process and wrote the first draft of the manuscript. All authors contributed to the manuscript revision, read, and approved the submitted version.

## Footnotes

^1^ https://huggingface.co/sentence-transformers/msmarco-distilbert-base-v4

^2^The architecture of BioBERT is similar to that of BERT base, with 12 transformer blocks. The input to BioBERT is a sequence of tokens, which are first passed through an embedding layer to convert them into fixed-size vectors. The embeddings are then fed into the transformer blocks, which consist of self-attention and feed-forward layers. The self-attention layer allows the model to capture contextual relationships between the tokens in the input sequence, while the feed-forward layer enables the model to learn complex representations of the input (https://github.com/dmis-lab/biobert).

^3^In this paper, we used the same values employed in Upadhyay et al. (2022), which produced the best evaluation results in ranking documents.

^4^For finding the optimal *w*_*d*_ value, we performed a grid search using 5 queries (randomly selected) and document related to those queries. The grid search involved systematically testing different values of *w*_*d*_ within a predefined range, and evaluating the performance of the system for each value of *w*_*d*_ using a predefined set of metrics (F1). The aim of this process was to identify the value of *w*_*d*_ that yielded the best performance in terms of the selected metrics, and therefore the best overall performance for the system.

^5^ https://commoncrawl.org/2016/10/news-dataset-available/

^6^In fact, in the TREC “Health Misinformation Track” (Clarke et al., 2020), documents were labeled by human assessors; in this sense, they were able to assess the *credibility* of documents, that is, the extent to which one perceives information to be believable (McKnight and Kacmar, 2007). This concept is not totally overlapping with that of truthfulness, although when labeling, human assessors were told to rely on criteria that were as objective as possible.

^7^PyTerrier is a Python-based retrieval framework for simple and complex Information Retrieval pipelines by making use of the Terrier IR platform for basic document indexing and retrieval (https://github.com/terrier-org/pyterrier).

^8^ https://huggingface.co/dmis-lab/biobert-v1.1

^9^ https://www.nltk.org/

^10^ https://anvil.works/

^11^We are aware that these results are still far from optimal, especially in the sensitive context of identifying misinformation in the medical field. However, this is not the main purpose of the article, which is focused on illustrating the explainability of these results to the user so that they can make their own decisions.

^12^The T5 model is a pre-trained language model developed by Google that uses a Transformer-based architecture to generate text (Raffel et al., 2020). It was trained on a large corpus of text using a task-specific approach called “text-to-text” learning, where the model learns to perform a specific Natural Language Processing task by mapping input text to output text. For our title generation task, we used the T5 implementation provided at the following address: https://huggingface.co/fabiochiu/t5-small-medium-title-generation.

^13^The questionnaire template provided to human assessors is available at the following address: https://rb.gy/ziqa3o.

## References

[B1] Adadi A. Berrada M. (2018). Peeking inside the black-box: a survey on explainable artificial intelligence (XAI). IEEE Access 6, 52138–52160. 10.1109/ACCESS.2018.2870052

[B2] Akerkar S. M. Kanitkar M. Bichile L. (2005). Use of the internet as a resource of health information by patients: a clinic-based study in the indian population. J. Postgrad. Med. 51, 116. 16006703

[B3] Anand A. Lyu L. Idahl M. Wang Y. Wallat J. Zhang Z. (2022). Explainable information retrieval: A survey. arXiv preprint arXiv:2211.02405.

[B4] Ayoub J. Yang X. J. Zhou F. (2021). Combat COVID-19 infodemic using explainable natural language processing models. Inform. Process. Manage. 58, 102569. 10.1016/j.ipm.2021.102569 33776192 PMC7980090

[B5] Bach S. Binder A. Montavon G. Klauschen F. Müller K.-R. Samek W. (2015). On pixel-wise explanations for non-linear classifier decisions by layer-wise relevance propagation. PLoS ONE 10, e0130140. 10.1371/journal.pone.0130140 26161953 PMC4498753

[B6] Bahkali S. Almaiman R. El-Awad M. Almohanna H. Al-Surimi K. Househ M. (2016). “Exploring the impact of information seeking behaviors of online health consumers in the arab world,” in Unifying the Applications and Foundations of Biomedical and Health Informatics (IOS Press), 279–282. 27350525

[B7] Bansal G. Wu T. Zhou J. Fok R. Nushi B. Kamar E.. (2021). “Does the whole exceed its parts? The effect of AI explanations on complementary team performance,” in Proceedings of the 2021 CHI Conference on Human Factors in Computing Systems, 1–16.

[B8] Bhaskara A. Skinner M. Loft S. (2020). Agent transparency: a review of current theory and evidence. IEEE Trans. Hum. Mach. Syst. 50, 215–224. 10.1109/THMS.2020.2965529

[B9] Bhatia P. Celikkaya B. Khalilia M. Senthivel S. (2019). “Comprehend medical: a named entity recognition and relationship extraction web service,” in 2019 18th IEEE International Conference On Machine Learning And Applications (ICMLA) (IEEE), 1844–1851.

[B10] Bjerring J. C. Busch J. (2021). Artificial intelligence and patient-centered decision-making. Philos. Technol. 34, 349–371. 10.1007/s13347-019-00391-633914464

[B11] Brachman R. J. Schmolze J. G. (1985). An overview of the KL-ONE knowledge representation system. Cogn. Sci. 9, 171–216.8591303

[B12] Brin S. Page L. (1998). The anatomy of a large-scale hypertextual web search engine. Comput. Netw. ISDN Syst. 30, 107–117.

[B13] Cabitza F. Ciucci D. Pasi G. Viviani M. (2022). Responsible AI in healthcare. arXiv preprint arXiv:2203.03616.

[B14] Cao W. Zhang X. Xu K. Wang Y. (2016). Modeling online health information-seeking behavior in China: the roles of source characteristics, reward assessment, and internet self-efficacy. Health Commun. 31, 1105–1114. 10.1080/10410236.2015.1045236 26861963

[B15] Chatila R. Dignum V. Fisher M. Giannotti F. Morik K. Russell S.. (2021). “Trustworthy AI,” in Reflections on Artificial Intelligence for Humanity, 13–39.

[B16] Chou W.-Y. S. Oh A. Klein W. M. (2018). Addressing health-related misinformation on social media. JAMA 320, 2417–2418. 10.1001/jama.2018.16865 30428002

[B17] Clarke C. L. Rizvi S. Smucker M. D. Maistro M. Zuccon G. (2020). “Overview of the trec 2020 health misinformation track,” in TREC.

[B18] Das B. Nirmala S. J. (2022). “Improving healthcare question answering system by identifying suitable answers,” in 2022 IEEE 2nd Mysore Sub Section International Conference (MysuruCon) (IEEE), 1–6.

[B19] Davagdorj K. Lee J. S. Pham V. H. Ryu K. H. (2020). A comparative analysis of machine learning methods for class imbalance in a smoking cessation intervention. Appl. Sci. 10, 3307. 10.3390/app10093307

[B20] Di Sotto S. Viviani M. (2022). Health misinformation detection in the social web: an overview and a data science approach. Int. J. Environ. Res. Public Health 19, 2173. 10.3390/ijerph19042173 35206359 PMC8872515

[B21] Eurostat (2022). EU Citizens: Over Half Seek Health Information Online. Technical report, Eurostat. Available online at: https://ec.europa.eu/eurostat/web/products-eurostat-news/-/edn-20220406-1 (accessed February 28, 2023).

[B22] Eysenbach G. (2007). From intermediation to disintermediation and apomediation: new models for consumers to access and assess the credibility of health information in the age of web2. 0. Stud. Health Technol. Inform. 129, 162. 17911699

[B23] Ferrara E. (2019). The history of digital spam. Commun. ACM 62, 82–91. 10.1145/3299768

[B24] Fleiss J. L. (1971). Measuring nominal scale agreement among many raters. Psychol. Bull. 76, 378.

[B25] Fox S. Duggan M. (2013). Health Online 2013. Technical report, Pew Research Center. Available online at: http://www.pewinternet.org/2013/01/15/health-online-2013/ (accessed February 28, 2023).

[B26] Gedikli F. Jannach D. Ge M. (2014). How should I explain? a comparison of different explanation types for recommender systems. Int. J. Hum. Comput. Stud. 72, 367–382. 10.1016/j.ijhcs.2013.12.007

[B27] Graham S. Brookey J. (2008). Do patients understand? Permanente J. 12, 67. 10.7812/TPP/07-144 21331214 PMC3037129

[B28] Guidotti R. Monreale A. Ruggieri S. Turini F. Giannotti F. Pedreschi D. (2018). A survey of methods for explaining black box models. ACM Comput. Surv. 51, 1–42. 10.1145/3236009

[B29] Gunning D. Stefik M. Choi J. Miller T. Stumpf S. Yang G.-Z. (2019). XAI-explainable artificial intelligence. Sci. Robot. 4, eaay7120. 10.1126/scirobotics.aay7120 33137719

[B30] Harris G. (2021). Combating the spread of health misinformation on social media. Brit. J. Healthcare Manage. 27, 40–42. 10.12968/bjhc.2020.0128

[B31] Inam R. Terra A. Mujumdar A. Fersman E. Vulgarakis A. (2021). Explainable AI – How Humans Can Trust AI.

[B32] Islam M. R. Liu S. Wang X. Xu G. (2020). Deep learning for misinformation detection on online social networks: a survey and new perspectives. Soc. Netw. Anal. Mining 10, 1–20. 10.1007/s13278-020-00696-x 33014173 PMC7524036

[B33] Kindig D. A. Panzer A. M. Nielsen-Bohlman L. (eds.). (2004). Health Literacy: A Prescription to End Confusion. Washington, DC: National Academies Press. 25009856

[B34] Kou Z. Shang L. Zhang Y. Wang D. (2022). HC-COVID: a hierarchical crowdsource knowledge graph approach to explainable COVID-19 misinformation detection. Proc. ACM Hum. Comput. Interact. 6, 1–25. 10.1145/349285537360538

[B35] Kou Z. Zhang D. Y. Shang L. Wang D. (2020). “Exfaux: a weakly-supervised approach to explainable fauxtography detection,” in 2020 IEEE International Conference on Big Data (Big Data) (IEEE), 631–636.

[B36] Lee J. Yoon W. Kim S. Kim D. Kim S. So C. H. Kang J. (2020). BioBERT: a pre-trained biomedical language representation model for biomedical text mining. Bioinformatics 36, 1234–1240. 10.1093/bioinformatics/btz682 31501885 PMC7703786

[B37] Liu N. Hu Q. Xu H. Xu X. Chen M. (2021). MED-BERT: a pretraining framework for medical records named entity recognition. IEEE Trans. Indus. Inform. 18, 5600–5608. 10.1109/TII.2021.3131180

[B38] Lu Y.-J. Li C.-T. (2020). GCAN: graph-aware co-attention networks for explainable fake news detection on social media. arXiv preprint arXiv:2004.11648.

[B39] Lundberg S. M. Lee S.-I. (2017). “A unified approach to interpreting model predictions,” in Advances in Neural Information Processing Systems 30.

[B40] Markus A. F. Kors J. A. Rijnbeek P. R. (2021). The role of explainability in creating trustworthy artificial intelligence for health care: a comprehensive survey of the terminology, design choices, and evaluation strategies. J. Biomed. Inform. 113, 103655. 10.1016/j.jbi.2020.103655 33309898

[B41] Marsden C. Meyer T. (2019). Regulating Disinformation with Artificial Intelligence: Effects of Disinformation Initiatives on Freedom of Expression and Media Pluralism. European Parliament.

[B42] McKnight D. H. Kacmar C. J. (2007). “Factors and effects of information credibility,” in Proceedings of the Ninth International Conference on Electronic Commerce, 423–432.

[B43] Merriam-Webster (ed.). (2023a). Factual. Available online at: https://www.merriam-webster.com/dictionary/factual (accessed October 03, 2023).

[B44] Merriam-Webster (ed.). (2023b). Misinformation. Available online at: https://www.merriam-webster.com/dictionary/misinformation (accessed October 03, 2023).

[B45] Merriam-Webster (ed.). (2023c). Truthful. Available online at: https://www.merriam-webster.com/dictionary/truthful (accessed October 03, 2023).

[B46] Miller T. (2019). Explanation in artificial intelligence: insights from the social sciences. Artif. Intell. 267, 1–38. 10.1016/j.artint.2018.07.007

[B47] Poerner N. Waltinger U. Schütze H. (2020). Inexpensive domain adaptation of pretrained language models: case studies on biomedical NER and COVID-19 QA. arXiv preprint arXiv:2004.03354.

[B48] Polley S. Janki A. Thiel M. Hoebel-Mueller J. Nuernberger A. (2021). “Exdocs: evidence based explainable document search,” in ACM SIGIR Workshop on Causality in Search and Recommendation.

[B49] Powell J. Inglis N. Ronnie J. Large S. (2011). The characteristics and motivations of online health information seekers: cross-sectional survey and qualitative interview study. J. Med. Internet Res. 13, e20. 10.2196/jmir.1600 21345783 PMC3221342

[B50] Pradeep R. Ma X. Nogueira R. Lin J. (2021). “VERA: prediction techniques for reducing harmful misinformation in consumer health search,” in Proceedings of the 44th International ACM SIGIR Conference on Research and Development in Information Retrieval, 2066–2070.

[B51] Qiao Y. Xiong C. Liu Z. Liu Z. (2019). Understanding the behaviors of bert in ranking. arXiv preprint arXiv:1904.07531.32818666

[B52] Raffel C. Shazeer N. Roberts A. Lee K. Narang S. Matena M.. (2020). Exploring the limits of transfer learning with a unified text-to-text transformer. J. Mach. Learn. Res. 21, 5485–5551.

[B53] Rahimi R. Kim Y. Zamani H. Allan J. (2021). Explaining documents' relevance to search queries. arXiv preprint arXiv:2111.01314.

[B54] Raval N. Verma M. (2020). One word at a time: adversarial attacks on retrieval models. arXiv preprint arXiv:2008.02197.

[B55] Ribeiro M. T. Singh S. Guestrin C. (2016). ““Why should I trust you?” Explaining the predictions of any classifier,” in Proceedings of the 22nd ACM SIGKDD International Conference on Knowledge Discovery and Data Mining, 1135–1144.

[B56] Rudin C. (2019). Stop explaining black box machine learning models for high stakes decisions and use interpretable models instead. Nat. Mach. Intell. 1, 206–215. 10.1038/s42256-019-0048-x 35603010 PMC9122117

[B57] Shin D. (2021). The effects of explainability and causability on perception, trust, and acceptance: implications for explainable AI. Int. J. Hum. Comput. Stud. 146, 102551. 10.1016/j.ijhcs.2020.102551

[B58] Shu K. Cui L. Wang S. Lee D. Liu H. (2019). “Defend: explainable fake news detection,” in Proceedings of the 25th ACM SIGKDD International Conference on Knowledge Discovery & Data Mining, 395–405.

[B59] Singh J. Anand A. (2019). “EXS: explainable search using local model agnostic interpretability,” in Proceedings of the Twelfth ACM International Conference on Web Search and Data Mining, 770–773.

[B60] Suominen H. Goeuriot L. Kelly L. Alemany L. A. Bassani E. Brew-Sam N.. (2021). “Overview of the clef ehealth evaluation lab 2021,” in Experimental IR Meets Multilinguality, Multimodality, and Interaction: 12th International Conference of the CLEF Association, CLEF 2021 (Springer), 308–323.

[B61] Tan S. S.-L. Goonawardene N. (2017). Internet health information seeking and the patient-physician relationship: a systematic review. J. Med. Internet Res. 19, e9. 10.2196/jmir.5729 28104579 PMC5290294

[B62] Thapa D. K. Visentin D. C. Kornhaber R. West S. Cleary M. (2021). The influence of online health information on health decisions: a systematic review. Patient Educ. Counsel. 104, 770–784. 10.1016/j.pec.2020.11.016 33358253

[B63] Tran J. Sellars M. Nolte L. White B. P. Sinclair C. Fetherstonhaugh D.. (2021). Systematic review and content analysis of australian health care substitute decision making online resources. Austral. Health Rev. 45, 317–327. 10.1071/AH20070 33472740

[B64] Upadhyay R. Pasi G. Viviani M. (2022). “An unsupervised approach to genuine health information retrieval based on scientific evidence,” in International Conference on Web Information Systems Engineering (Springer), 119–135.

[B65] Viviani M. Pasi G. (2017). Credibility in social media: opinions, news, and health information–a survey. Wiley Interdiscipl. Rev. Data Mining Knowledge Discov. 7, e1209. 10.1002/widm.1209

[B66] Yu P. Rahimi R. Allan J. (2022). “Towards explainable search results: a listwise explanation generator,” in Proceedings of the 45th International ACM SIGIR Conference on Research and Development in Information Retrieval, 669–680.

[B67] Zhou X. Zafarani R. (2020). A survey of fake news: fundamental theories, detection methods, and opportunities. ACM Comput. Surv. 53, 1–40. 10.1145/3395046

